# TSC22 domain family member 3 links natural killer cells to CD8+ T cell-mediated drug hypersensitivity

**DOI:** 10.1038/s41392-025-02300-0

**Published:** 2025-06-21

**Authors:** Lele Sun, Pengcheng Huai, Zhenzhen Wang, Qing Zhao, Yingjie Lin, Tingting Liu, Xiaotong Xue, Suiting Ao, Jiabao You, Yonghu Sun, Zihao Mi, Joshua Gardner, Paul J. Thomson, Dean J. Naisbitt, Xiaoli Meng, Jianjun Liu, Hong Liu, Furen Zhang

**Affiliations:** 1 Dermatology Hospital of Shandong First Medical University, Jinan, Shandong China; 2 Shandong Provincial Institute of Dermatology and Venereology, Shandong Academy of Medical Sciences, Jinan, Shandong China; 3 MRC Centre for Drug Safety Science, Department of Pharmacology and Therapeutic, Institute of Systems, Molecular and Integrative Biology, University of Liverpool, Liverpool, UK; 4 Laboratory of Human Genomics, Genome Institute of Singapore (GIS), Agency for Science, Technology and Research (A*STAR), Singapore, Republic of Singapore; Yong Loo Lin School of Medicine, National University of Singapore, Singapore, Republic of Singapore; 5 School of Public Health, Shandong First Medical University & Shandong Academy of Medical Sciences, Jinan, Shandong China; 6 Medical Science and Technology Innovation Center, Shandong First Medical University & Shandong Academy of Medical Sciences, Jinan, Shandong China; 7 Shandong University of Traditional Chinese Medicine, Jinan, Shandong China

**Keywords:** Innate immunity, Innate immunity

## Abstract

Severe cutaneous adverse drug reactions (SCARs) are life-threatening diseases, which are associated with *human leukocyte antigen* (*HLA*) risk variants. However, the low positive predictive values of *HLA* variants suggest additional factors influence disease susceptibility. Using dapsone hypersensitivity syndrome (DHS) as a paradigm for SCARs, we show that the DHS patients harbor a sex-related global reduction in blood NK cells, contributing to the higher incidence of reactions in females. Single-cell RNA sequencing revealed a decrease in the immunoregulatory *CD56*^low^ *XCL1/2*^low^ NK cell subset and an expansion of *CD56*^high^ *XCL1/2*^high^ NK cell subsets with an effector phenotype in DHS patients compared to dapsone-tolerant individuals. Functionally, interleukin-15 superagonist-induced activation of NK cells exacerbated SCARs-like symptoms in a murine model. Mechanistically, TSC22 domain family member 3 (TSC22D3) deficiency enhanced NK cell effector function, shifting the immune response from CD4+ T cell to CD8+ T cell function. These results demonstrate that TSC22D3-regulated NK cells play a critical role in predisposing to drug hypersensitivity reactions, bridging innate and adaptive immune dysregulation in SCARs pathogenesis. Our study highlights the importance of NK cell heterogeneity and TSC22D3 in immune-mediated hypersensitivity disorders, offering potential therapeutic targets for SCARs and related conditions.

## Introduction

Severe cutaneous adverse drug reactions (SCARs) constitute a spectrum of life-threatening, T cell-mediated delayed hypersensitivity disorders characterized by heterogeneous clinical manifestations and significant mortality rates. These reactions are clinically classified into three major subtypes: acute generalized exanthematous pustulosis (AGEP), exhibiting a mortality rate of less than 5%; drug-induced hypersensitivity syndrome (DIHS), also known as drug reaction with eosinophilia and systemic symptoms (DRESS) or drug induced delayed multiorgan hypersensitivity syndrome (DIDMOHS), with approximately 10% mortality rate; and Stevens-Johnson syndrome/toxic epidermal necrolysis (SJS/TEN), which demonstrates the highest mortality rate (5%-30%), depending on disease severity and extent of epidermal detachment.^1–3^ The strong genetic component of SCARs has been well-established through extensive pharmacogenetic studies identifying over 20 *human leukocyte antigen* (*HLA*) risk alleles, particularly HLA-B*13:01, HLA-B*15:02, HLA-B*57:01 and HLA-B*58:01, which show robust associations with SCARs pathogenesis.^4–7^ These discoveries have facilitated the clinical implementation of preemptive pharmacogenetic screening strategies.^4–7^ However, the translational application of these genetic markers remains constrained by their characteristically low positive predictive values (PPVs; e.g., 3% for HLA-B*58:01 in allopurinol-induced SCARs and 7.8% for HLA-B*13:01 in dapsone hypersensitivity syndrome [DHS]). This highlights gaps in understanding SCARs pathophysiology, particularly how genetic susceptibility interacts with other immunological factors to determine clinical outcomes.

Current mechanistic frameworks have primarily focused on elucidating the mechanisms of adaptive immune dysregulation in SCARs pathogenesis, such as T cell receptor (TCR) clonotype, immunoregulation of CD4+ T cells, and antigen presentation machinery alterations. TCR sequencing analysis have identified specific TCR clonotypes preferentially expressed in blister fluid and blood of carbamazepine-induced SJS/TEN patients.^8^ Functionally, T cells carrying these TCR clonotypes triggered a carbamazepine specific immune response both in vitro and in HLA-B*15:02 transgenic mice.^8,9^ A study using HLA-B*57:01 transgenic mice indicated that deficiency of CD4+ T cells in vivo induced an abacavir hypersensitivity syndrome like phenotype. CD4+ T cells displayed an immunoregulatory phenotype, exerting an inhibitory effect on dendritic cells (DCs), which effectively controlled T cell activation.^10^ Our recent studies showed that the methylation level of transporter associated with antigen processing 2 (*TAP2*) was lower in DHS patients compared to dapsone tolerant individuals.^11^ Functional increase of TAP resulted in an enhanced capacity of antigen presenting cells to activate CD8+ T cells, which was regulated by tumor necrosis factor-alpha mediated inhibition of V-type immunoglobulin domain-containing suppressor of T-cell activation.^11,12^ Nevertheless, these findings fail to fully account for the discordance between HLA carrier status and clinical penetrance, suggesting unexplored contributions from innate immune regulators.

Emerging evidence positions natural killer (NK) cells as pivotal modulators bridging innate and adaptive immunity in hypersensitivity reactions through their heterogeneous subsets and remarkable functional plasticity. NK cells exert multifaceted immunoregulatory functions on adaptive immunity through major histocompatibility complex class II presentation, expression of several co-stimulatory molecules, such as OX40 ligand and CD86, and cytokine-mediated crosstalk with DCs, T cells, and B cells, thereby influencing Th1/Th2 polarization.^13,14^. Clinical observations reveal that perturbations in NK cell subsets correlate with dysregulated T cell responses in allergic disorders. Mechanistic studies reveal that NK cells from peanut-allergic individuals exhibit T cell-dependent activation and express both Th2 cytokines (IL-4, IL-13) and immunoregulatory cytokines (IL-10, TGF-β). This paradoxical cytokine signature suggests NK cells play a dual immunomodulatory role, both promoting and constraining type 2 inflammatory responses, in allergic diseases.^13^ In atopic dermatitis, reduced mature CD56^dim^ NK cell subsets coincide with exaggerated Th2 responses, highlighting the critical immunoregulatory function of NK cells in restraining type 2 inflammation.^14,15^ These observations have led to the compelling hypothesis that NK cell heterogeneity and functional plasticity may serve as crucial determinants of SCARs susceptibility by modulating the threshold for T cell activation and the subsequent inflammatory cascade.

Using DHS as a prototypical SCARs model, we identified striking NK cell repertoire skewing in peripheral blood mononuclear cells (PBMC) of DHS patients compared to dapsone-tolerant individuals. Epidemiological analyses revealed a striking female predominance in DHS and other adverse drug reaction incidences. This sex bias correlates with established sex differences in NK cell biology, suggesting a potential mechanistic basis for gender disparities in drug hypersensitivity. Single-cell RNA sequencing (scRNA-seq) delineated contracted *CD56*^low^ *XCL1/2*^low^ immunoregulatory NK subsets alongside expanded *CD56*^high^ *XCL1/2*^high^ effector populations in DHS patients. Notably, IL-15 superagonist treatment exacerbated SCARs-like pathology in murine models, implicating hyperactive NK effector responses in disease progression. Mechanistically, transcriptomic analyses uncovered systemic downregulation of TSC22 domain family member 3 (*TSC22D3*)—a negative regulator of NK cytotoxicity—in patient-derived NK cells. *TSC22D3* knockout enhanced NK cell-mediated CD8+ T cell priming through cytokine-dependent and contact-dependent pathways while impairing proliferative capacity, mirroring the hypoproliferative yet hyperinflammatory NK phenotype observed in DHS. Collectively, these findings establish NK cells as non-canonical but crucial determinants of SCARs pathogenesis and potential therapeutic targets for immune-mediated hypersensitivity disorders. Future research directions should focus on elucidating the precise molecular mechanisms governing NK cell-T cell interactions in SCARs and exploring targeted therapeutic strategies to restore immune homeostasis in these devastating conditions.

## Results

### Different composition of immune cells between DHS patients and dapsone-tolerant individuals

**Fig. 1:**
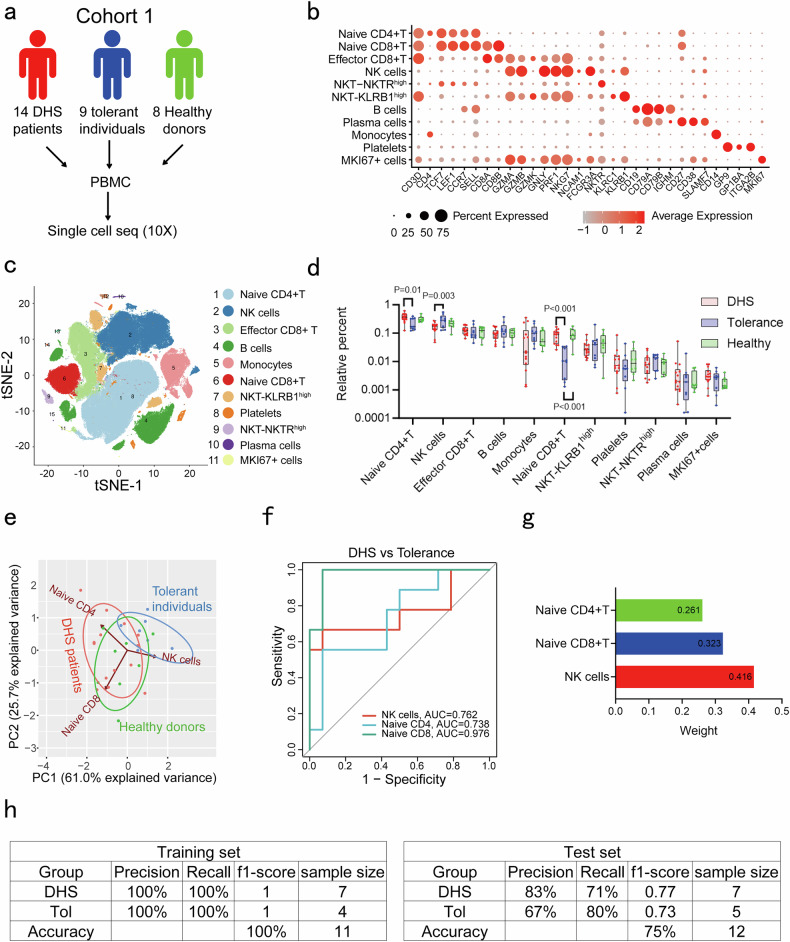
Different composition of immune cells between DHS patients and dapsone tolerant individuals. **a** Flow chart of scRNA-seq in cohort 1. PBMC of 14 DHS patients, nine dapsone-tolerant individuals and eight healthy donors were subject to scRNA-seq (without stimulation). **b** 11 distinct clusters representing different cell types in PBMC were identified based on the expression of cluster-specific markers and canonical signature genes such as *CD3D*, *CD4*, *CD8A*, *CD14*, *CD19* and *CD56* (*NCAM1*). **c** t-SNE plot displaying 11 identified distinct cellular clusters. **d** Relative abundances of each cluster in DHS patients, dapsone-tolerant individuals and healthy donors. One-way ANOVA followed by LSD was used for statistical analysis. **e** Principal component analyses (PCA) were performed using naive CD4+ T, naive CD8+ T and NK cells. The first and second principal components indicated the percentage of the variance. Each point represents one sample. **f** A receiver operating characteristic (ROC) curve showed the area under the curve (AUC), sensitivity and specificity of naive CD4+ T, naive CD8+ T and NK cells in distinguishing DHS patients from dapsone-tolerant individuals. **g** RF model showing the variable importance (weight) of naive CD4+ T, naive CD8+ T and NK cells in distinguishing DHS patients from dapsone-tolerant individuals. **h** RF model showing the accuracy in the training set and testing set, respectively

A comprehensive analysis of immune cell profiles using scRNA-seq was performed using peripheral blood mononuclear cells (PBMC) of 14 DHS patients carrying *HLA* risk variant HLA-B*13:01, nine dapsone-tolerant individuals carrying HLA-B*13:01 and eight healthy donors not carrying HLA-B*13:01 (Cohort 1) (Fig. 1a-c). Supplementary Table 1 shows clinical characteristics of the DHS patients, dapsone patch testing and enzyme-linked immunospot assay (ELISPOT) results, which have been previously described.^7,16^ Comparison of relative abundances of immune cell subsets in the three groups identified three significant cell subsets (naive CD4+ T, naive CD8+ T and NK cells) and the difference was mainly displayed between DHS patients and dapsone-tolerant individuals (*P* < 0.05, unpaired t-test) (Fig. 1d). This likely reflects the fact that DHS and tolerance represent opposite ends of the clinical spectrum, while healthy donors represent a mixed population that could theoretically include both potential DHS patients and tolerant individuals if exposed to dapsone. DHS patients exhibited an increase of naive CD4+ T and naive CD8+ T and a reduction of NK cells compared to dapsone-tolerant individuals (Fig. 1d). scRNA-seq data further demonstrated that these expanded naive T cell populations in DHS patients displayed elevated JAK-STAT pathway activity (*STAT1*) (Supplementary Fig. 1), a pathway critical for SCARs progression.^17,18^ This pre-activation state of naive T cells suggests an enhanced propensity for effector differentiation, which may contribute to the dysregulated immune response characteristic of DHS. An unsupervised principal component analyses (PCA) indicated that these three subsets accounted for 86.7% of the variance in the data set (Fig. 1e). A receiver operating characteristic (ROC) curve showed that all these three subsets can effectively distinguish DHS patients from dapsone-tolerant individuals (Fig. 1f).

To further validate the performance of these three subsets in distinguishing DHS patients from dapsone-tolerant individuals, a second model, random forest (RF) analysis, was constructed. The proportion of training set was set at 50%, and testing set was used to define model accuracy and predictive performance. The variable importance (weight) showed NK cells and naive CD8+ T cells were the first and the second most important variable for the classification (Fig. 1g), respectively. RF model distinguished DHS patients from dapsone-tolerant individuals with an accuracy of 100% and 75% in training sets and testing sets, respectively (Fig. 1h).

### Higher incidence of DHS in females is associated with lower NK cells number

**Fig. 2:**
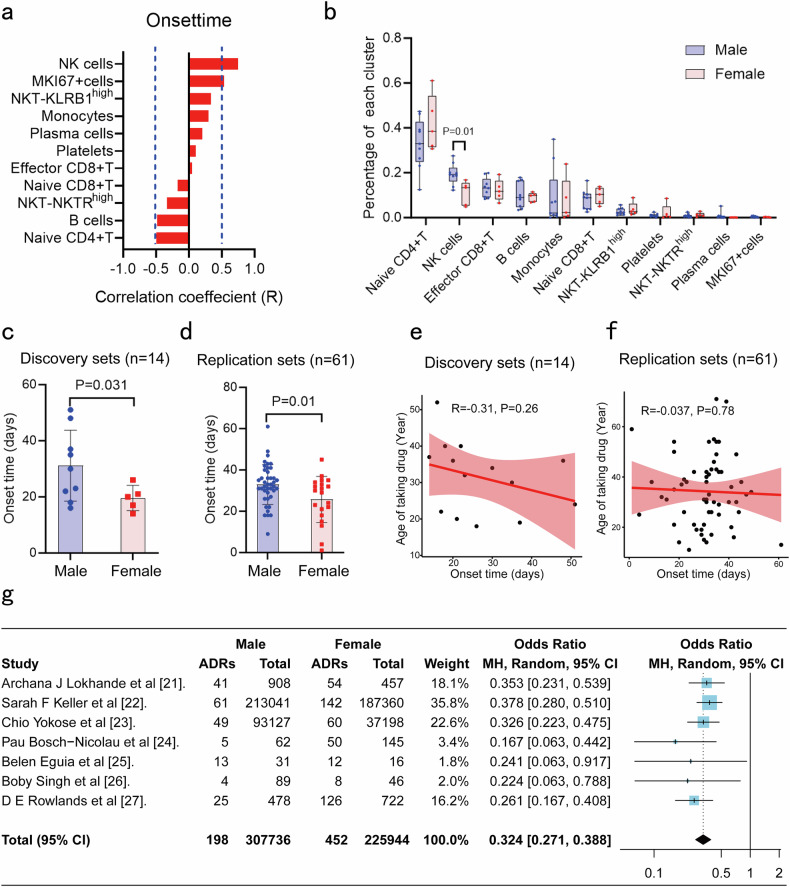
Higher incidence of DHS in female was associated with the lower NK cells. **a** Correlation analysis between percentage of each cluster in total PBMC and the onset time of DHS (from taking dapsone to onset). **b** Comparison of percentage of each cluster in total PBMC between male and female was performed in DHS patients. **c**, **d** The onset time of DHS between male and female was compared in discovery sets from cohort 1 (*n* = 14) (**c**) and separate replication sets (*n* = 61) (**d**). **e**, **f** Correlation analysis between age of taking dapsone and the onset time of DHS was performed in discovery sets from cohort 1 (*n* = 14) (**e**) and separate replication sets (*n* = 61) (**f**). **g** Meta-analysis enrolled a total of 533,680 patients taking drugs (307,736 were male and 225,944 were female) from seven studies was performed to validate the common sex difference in development of ADRs. The unpaired two-sided student’s *t*-test was used for statistical analysis

Correlation analysis indicated that the percentage of NK and *MKI67*^high^ cells in PBMC showed a significantly positive correlation with onset time of DHS (from commencing dapsone to onset), especially for NK cells (R = 0.75, P = 0.002) (Fig. 2a). These observations suggested that NK cells play an important role in pathogenesis of DHS. Furthermore, the percentage of NK cell subsets in PBMC of male DHS patients was higher (*P* = 0.01, unpaired t-test) (Fig. 2b) when male and female DHS patients were compared. This is consistent with recent studies that a reported higher percentage of NK cells in males.^19,20^ From these observations, we hypothesized that the onset time of DHS would differ between male and female. As expected, the onset time of DHS in males was significant longer than that of females in the cohort 1 discovery set (*n* = 14, *P* = 0.031, unpaired t-test) and a separate replication set from our previous study^6^ (*n* = 61, *P* = 0.01, unpaired t-test) (Fig. 2c, d). We also tested whether age at the time of dapsone administration influenced the onset time of DHS; however, no association was observed (Fig. 2e, f), excluding age as a parameter for DHS susceptibility.

On the basis of the above results, we speculated that the incidence of DHS was different between male and female. To test this, we performed a retrospective review and calculated the incidence of DHS between male and female in leprosy patients treated with dapsone from 2008 to 2012 in China (Data was provided by National Center for STD and Leprosy Control). The incidence of DHS in female was significantly higher than that of male (1.7%, 35/2020 vs 0.8%, 42/4865, *P* = 0.004, chi-square test), which was consistent with the lower percentage of NK cells and shorter onset time in female. Additionally, to further validate the common sex difference in development of adverse drug reactions (ADRs), we performed meta-analysis on the incidence of ADRs caused by other drugs between male and female.^21–27^ A total of 533,680 patients (307,736 were male and 225,944 were female) were included from seven studies, of whom 650 patients developed ADRs (198 were male and 452 were female). These ADRs were strongly linked to sex differences, in which the incidence of ADRs in male (0.06%) were significantly lower than that of female (0.2%) (OR 0.324, 95% confidence interval [CI] 0.271-0.388) (Fig. 2g). These results suggested a common sex difference in the risk of ADRs, with an increased susceptibility in females.

### The dapsone-dependent NK cell response is greater in DHS patients than dapsone-tolerant individuals

**Fig. 3:**
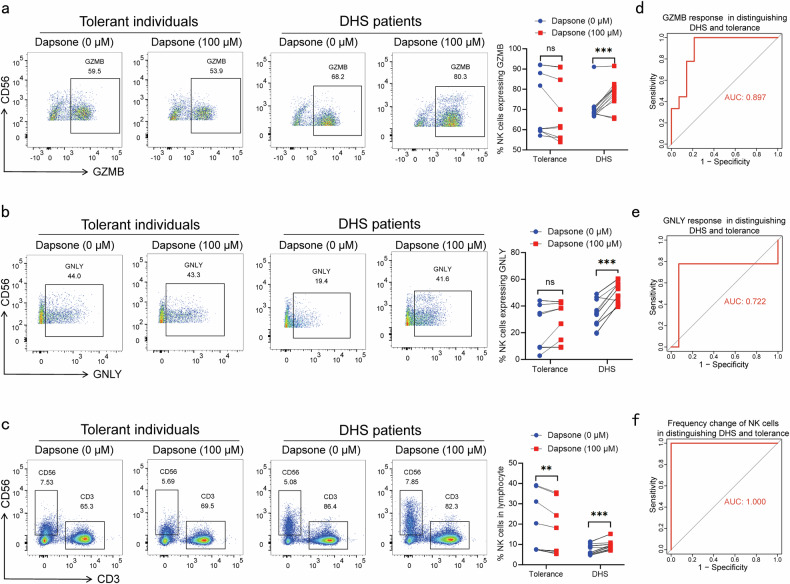
The dapsone dependent response in NK cells was greater in DHS patients than dapsone tolerant individuals. PBMC from 14 DHS patients and nine dapsone tolerant individuals were cultured with medium or dapsone (100 µM) for six days, and the percentage of activated NK cells that expressed GZMB (**a**) and GNLY (**b**) and the frequency of NK cells in total lymphocytes (**c**) were analyzed by flow cytometry. ROC curve showed the AUC, sensitivity and specificity of GZMB response (**d**), GNLY response (**e**) and frequency change of NK cells in lymphocytes (**f**) in distinguishing DHS patients from dapsone-tolerant individuals. The paired two-sided student’s t-test was used for statistical analysis (**P < 0.01 and ***P < 0.001)

Although NK cells are innate cells, they share a number of characteristics with T cells by secreting the similar effector molecules, such as granzyme B (GZMB) and granulysin (GNLY).^14^ To examine whether NK cells could be activated with dapsone, PBMC from 14 DHS patients and nine dapsone-tolerant individuals were exposed to the drug for 6 days and NK effector molecules were analyzed using flow cytometry. In comparison with medium control group, both the percentage and median fluorescence intensity (MFI) of activated NK cells that expressed GZMB and GNLY increased significantly following dapsone stimulation in DHS patients (*P* < 0.05, paired t-test) (Fig. 3a, b, d, e and Supplementary Fig. 2). However, a significant increase in NK effector molecules was not observed in dapsone-tolerant individuals (*P* > 0.05, paired t-test) (Fig. 3a, b, d, e and Supplementary Fig. 2). These findings are consistent with a previous study, which showed that NK cells are activated by peanut allergen in peanut allergic patients but not nonallergic participants.^13^

In addition to effector molecules expression, 13 out of 14 DHS patients (92.9%) showed a significant increase in the frequency of NK cells in lymphocytes following dapsone stimulation (*P* < 0.05, paired t-test) (Fig. 3c, f). Unexpectedly, all dapsone-tolerant individuals (100%, *P* < 0.05, paired t-test) showed significant decrease in frequency of NK cells in lymphocytes following dapsone stimulation (*P* < 0.05, paired t-test) (Fig. 3c, f). Collectively, these observations suggest that NK cells act as effector cells on the setting of DHS.

### NK cell subsets exhibit immune effector and immunoregulatory phenotypes

**Fig. 4:**
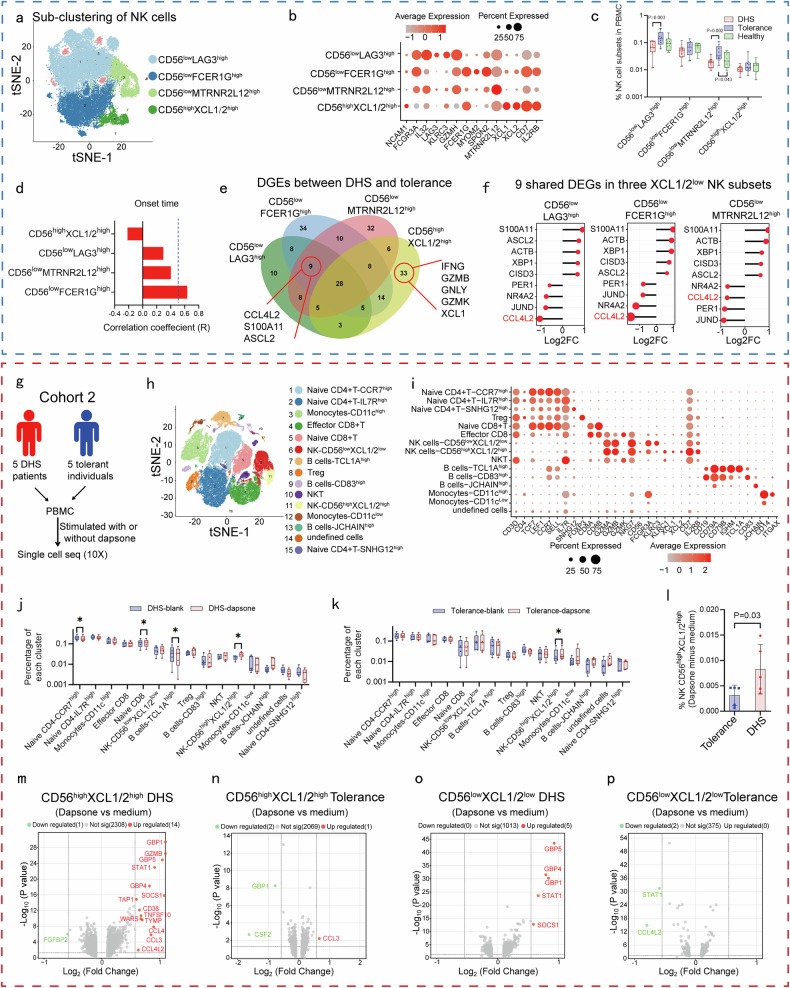
NK cell subsets exhibit immune effector and immunosuppressive phenotypes. **a** Sub-clustering analysis for NK cells from cohort 1. t-SNE plot showed the main four NK cell subsets, including *CD56*^low^ *LAG3*^high^, *CD56*^low^ *FCER1G*^high^, *CD56*^low^ *MTRNR2L12*^high^ and *CD56*^high^ *XCL1/2*^high^ NK cell subsets. **b** Bubble diagram indicated the expression of cluster-specific markers. **c** Relative abundances of each NK cluster in DHS patients, dapsone-tolerant individuals and healthy donors. One-way ANOVA followed by LSD was used for statistical analysis. **d** Correlation analysis between percentage of each NK cluster in total PBMC and the onset time of DHS. **e** The Venn diagram revealed the sharing and specific DEGs comparing between DHS patients and dapsone-tolerant individuals in *CD56*^low^ *LAG3*^high^, *CD56*^low^ *FCER1G*^high^, *CD56*^low^ *MTRNR2L12*^high^ and *CD56*^high^ *XCL1/2*^high^ NK cell subsets. **f** The fold change value of the shared genes in the other three *CD56*^low^ *XCL1/2*^low^ NK cell subsets between DHS patients and dapsone-tolerant individuals. **g** Flow chart of scRNA-seq in cohort 2. PBMC of five DHS patients and five dapsone-tolerant individuals were stimulated with or without dapsone (100 µM) for four days and subject to scRNA-seq. **h**, **i** 15 distinct clusters representing different cell types in PBMC were identified based on the expression of cluster-specific markers and canonical signature genes such as *CD3D*, *CD4*, *CD8A*, *CD14*, *CD19* and *CD56* (*NCAM1*). **j**, **k** Relative abundances of each cluster was compared between stimulated with or without dapsone in DHS patients (**j**) and dapsone-tolerant individuals (**k**), respectively. The paired two-sided student’s *t*-test was used for statistical analysis (*P < 0.05). **l** The abso**l**ute elevation of frequency of *CD56*^high^ *XCL1/2*^high^ NK cells subsets in total PBMC after dapsone stimulation in DHS patients and dapsone-tolerant individuals. The unpaired two-sided student’s *t*-test was used for statistical analysis. **m–p** Volcano plot revealed distinct transcriptional profiles in *CD56*^high^ *XCL1/2*^high^ and *CD56*^low^ *XCL1/2*^low^ NK cells subsets between stimulating with or without dapsone in DHS patients and dapsone-tolerant individuals. Blue dotted frame, cohort 1; Red dotted frame, cohort 2

Lower abundances of NK cells but higher response to dapsone stimulation in DHS patients compared to dapsone-tolerant individuals seem somewhat paradoxical. However, NK cells comprise different subsets and show a large degree of phenotypic heterogeneity not only exerting cytotoxic activity but also playing a role in immune regulation.^14,15^ Therefore, we hypothesized that NK cell subsets might be differential in DHS patients and dapsone-tolerant individuals. To examine this, we first performed sub-clustering analysis for NK cells from cohort 1. The sub-clustering of NK cells identified seven subsets, including *CD56*^low^ *LAG3*^high^ NK cell subsets, *CD56*^low^ *FCER1G*^high^ NK cell subsets, *CD56*^low^ *MTRNR2L12*^high^ NK cell subsets, *CD56*^high^ *XCL1/2*^high^ NK cell subsets and other three subsets which were not analyzed due to the majority of individuals did not expressing them (Fig. 4a, b and Supplementary Fig. 3). *CD56*^low^ *LAG3*^high^ NK cell subset was marked by low expression of *CD56* (*NCAM1*) and high expression of *CD16* (*FCGR3A*), *IL32*, *LAG3*, *KLRC3* and *GZMH*. *CD56*^low^ *FCER1G*^high^ NK cell subset was marked by low expression of *CD56* and high expression of *CD16*, *FCER1G*, *MYOM2* and *SPON2*. *CD56*^low^ *MTRNR2L12*^high^ NK cell subset was marked by low expression of *CD56* and high expression of *CD16* and *MTRNR2L12. CD56*^high^ *XCL1/2*^high^ NK cell subset was marked by low expression of *CD16* and high expression of *CD56*, *XCL1*, *XCL2*, *CD7* and *IL2RB*. These four NK subsets correspond with NK3, NK1C, NK1B and NK2 subsets based on the transcriptional profile in the most recent NK phenotyping study by Rebuffet et al.^28^ Of the four consistently-expressed NK cell subsets, DHS patients exhibited a selective reduction in *CD56*^low^ *LAG3*^high^ NK and *CD56*^low^ *MTRNR2L12*^high^ cells (*P* < 0.05, unpaired t-test) (Fig. 4c), demonstrating that these subsets contribute towards the global reduction of NK cells in DHS patients.

To analyze the phenotypic difference between the four NK cell subsets, correlation analysis between the onset time of DHS and percentage of each NK cell subset in total PBMC was performed. *CD56*^high^ *XCL1/2*^high^ NK cells correlate negatively with onset time (promoting DHS), while *CD56*^low^ *XCL1/2*^low^ subsets correlate positively (delaying DHS) (Fig. 4d). To identify cellular programs that may underlie the distinct phenotype of NK cell subsets, we analyzed differentially expressed genes (DEGs) between DHS patients and dapsone-tolerant individuals from cohort 1 in the four NK cell subsets. The Venn diagram revealed *CD56*^high^ *XCL1/2*^high^ NK cell subsets of DHS patients specifically up-regulated expression of effector molecules, such as *IFN*-γ, *GZMB*, *GZMK, GNLY* and *XCL1* compared to dapsone-tolerant individuals (Fig. 4e). These results suggested that *CD56*^high^ *XCL1/2*^high^ NK cell subsets showed a more effector phenotype in DHS patients potentially contributing to the development of DHS. In contrast, among the shared genes of the other three *CD56*^low^ *XCL1/2*^low^ NK cell subsets, C-C motif chemokine ligand 4 like 2 (*CCL4L2*), which is a well-known T cell activator,^29,30^ showed a significant down-regulation in DHS patients compared to dapsone-tolerant individuals (Fig. 4f). These results suggest that the other three *CD56*^low^ *XCL1/2*^low^ NK cell subsets have a negative feedback regulation on T cells through regulating the expression of *CCL4L2*.

To validate whether *CD56*^high^ *XCL1/2*^high^ NK cell subsets act as effector cells after dapsone stimulation in DHS patients, scRNA-seq was performed with PBMC from both DHS patients (*n* = 5) and dapsone-tolerant individuals (*n* = 5) (cohort 2) after treatment with dapsone or medium alone for four days (Fig. 4g–i). All these subjects derived from cohort 1 (Supplementary Table 1). Based on the expression of established and specific markers, main 15 cell clusters were identified (Fig. 4h, i). Among these cell clusters, *CCR7*^high^ naive CD4+ T cell subsets exhibited a reduction and naive CD8+ T, *TCL1A*^high^ B and *CD56*^high^ *XCL1/2*^high^ NK cell subsets exhibited an increase in DHS patients after dapsone stimulation (*P* < 0.05, paired t-test) (Fig. 4j). Although dapsone-tolerant individuals also displayed elevated frequencies of *CD56*^high^ *XCL1/2*^high^ NK cell subsets after dapsone stimulation (Fig. 4k), this elevation was significantly lower than DHS patients (*P* = 0.03, unpaired t-test) (Fig. 4l). These data are similar to the flow cytometry analysis that the frequency of total NK cells in lymphocytes is increased in DHS patients (*P* < 0.05, paired t-test), but slightly decreased in dapsone-tolerant individuals (*P* < 0.05, paired t-test), after dapsone stimulation (Fig. 3c).

Paired comparisons of DEGs in *CD56*^high^ *XCL1/2*^high^ and *CD56*^low^ *XCL1/2*^low^ NK cell subsets between dapsone and medium stimulation revealed distinct transcriptional profiles between DHS patients and dapsone-tolerant individuals’ NK cell subsets, with an increased number of DEGs in DHS patients (Fig. 4m–p). As expected, the *CD56*^high^ *XCL1/2*^high^ NK cell subset showed a significant increase in effector molecules such as *GZMB*, JAK/STAT and interferon signaling pathway molecules (*STAT1*, *GBP1* and *GBP5*) after dapsone stimulation in DHS patients but not dapsone-tolerant individuals (Fig. 4m, n). This result was consistent with *GZMB* the specific up-regulation in *CD56*^high^ *XCL1/2*^high^ NK cell subsets of DHS patients compared to dapsone-tolerant individuals observed in cohort 1 (Fig. 4e). These findings further demonstrated that *CD56*^high^ *XCL1/2*^high^ NK cells serve as effector cells in DHS patients. Additionally, *CD56*^low^ *XCL1/2*^low^ NK cell subsets of dapsone-tolerant individuals showed a significant decrease of *CCL4L2* after dapsone stimulation (Fig. 4o, p), suggesting *CD56*^low^ *XCL1/2*^low^ NK cell subsets have a greater immunoregulatory capacity in dapsone-tolerant individuals.

Collectively, *CD56*^high^ *XCL1/2*^high^ NK cell subsets exhibited a more immune effector phenotype in DHS patients compared to dapsone-tolerant individuals, while the *CD56*^low^ *XCL1/2*^low^ NK cell subsets with an immunoregulatory phenotype were decreased in DHS patients. Both of the altered composition and function of NK cell subsets contribute towards the development of DHS.

### Effector NK cells aggravated the SCARs like symptoms in established mice model of SCARs

**Fig. 5:**
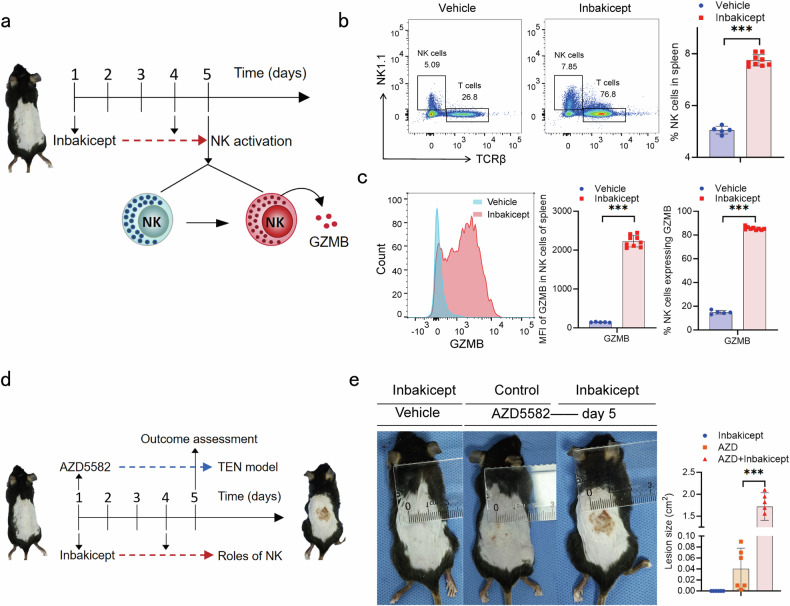
Effector NK cells aggravated the SCARs like symptoms in established mice model of SCARs. **a** C57BL/6 mice were injected intraperitoneally with 100 μl of 0.2 mg/ml inbakicept (IL-15 superagonist) on day one and day four to induce effector NK cells activation. **b**, **c** On day five, mice were euthanized and splenic cells were collected. The frequency of NK cells in total splenic cells (**b**) and the percentage and median fluorescence intensity (MFI) (**c**) of NK cells that expressed GZMB were analyzed by flow cytometry. **d** C57BL/6 mice were injected subcutaneously with 100 μl of 1 mg/ml AZD5582 on day one and injected intraperitoneally with 100 μl of 0.2 mg/ml inbakicept (IL-15 superagonist) on day one and day four. **e** On day five, mice were euthanized and the sites were photographed and scored. The unpaired two-sided student’s *t*-test was used for statistical analysis (***P < 0.001)

Based on the findings of *CD56*^high^ *XCL1/2*^high^ NK cell subsets with effector phenotype in DHS patients, we next evaluated the impact of activated effector NK cells for the development of SCARs in the mouse model of SCARs. IL-15 superagonist and antagonist of inhibitor of apoptosis proteins (IAP) are established methods to activate the effector function of NK cells and induce SCARs-like disease in mice, respectively.^15,18^ As expected, after two intraperitoneal injections of inbakicept (IL-15 superagonist) (Day one and day four), both the percentage of total NK cells and effector NK cells that expressed GZMB increased significantly (*P* < 0.05, t-test) (Fig. 5a–c). These results validated the effector NK cell subsets were activated by IL-15 superagonist in vivo. And, after one subcutaneous injection of AZD5582 (antagonist of IAP), the mice developed significant TEN-like cutaneous symptom (cutaneous inflammation and histological features of TEN) on day five (Fig. 5d, e and Supplementary Fig. 4). Importantly, when combined administration of AZD5582 and inbakicept, the mice developed a more significant TEN-like cutaneous symptom on day five than AZD5582 alone (Fig. 5d, e and Supplementary Fig. 4). These results validated the activated effector NK cells could contribute toward the development of SCARs, notwithstanding that this murine model does not fully capture drug-specific hypersensitivity.

### *TSC22D3* governed NK cell phenotype

**Fig. 6:**
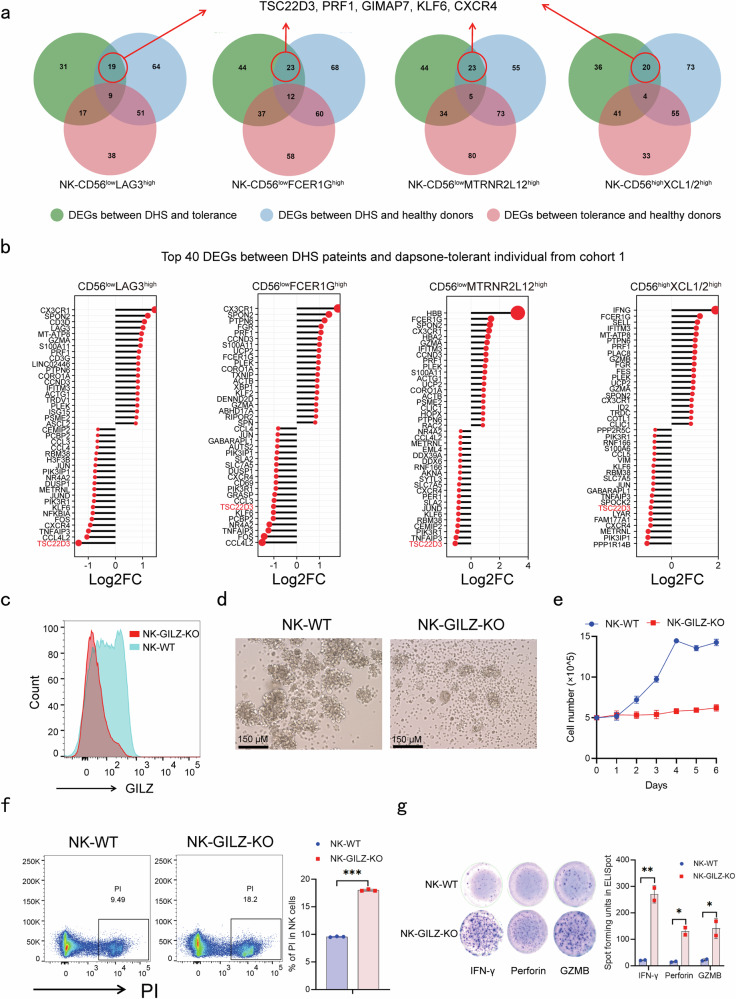
TSC22D3 governed NK cell phenotype. **a** The Venn diagram revealed the sharing DEGs between groups of DHS patients compared with dapsone-tolerant individuals and DHS patients compared with healthy donors, but not in group of dapsone-tolerant individuals compared with healthy donors in each NK cell subset. **b** The top 40 DEGs in each NK cell subset between DHS patients and dapsone-tolerant individuals from cohort 1. **c** The expression of GILZ (*TSC22D3* encoded protein) was detected by flow cytometry in *TSC22D3* knockout and wild-type (WT) NK cell lines (NK-92MI). **d** Bright-field microscopy images showed the morphology of *TSC22D3* knockout and wild-type (WT) NK cell lines. **e** Cell growth curves of *TSC22D3* knockout and WT NK-92MI. **f** Cell death was detected using propidine iodide (PI) by flow cytometry in *TSC22D3* knockout and WT NK-92MI. **g** 3 × 10^3 *TSC22D3* knockout or WT NK 92MI were plated in 96 well ELISPOT plate and cultured for one day. The release of IFN-γ, perforin and GZMB was detected by ELISPOT assay. The unpaired two-sided student’s *t*-test was used for statistical analysis (*P < 0.05,**P < 0.01 and ***P < 0.001)

To explore the key factors that govern the varied phenotype of NK cell subsets, DEGs in NK cell subsets between DHS patients, dapsone-tolerant individuals and healthy donors from cohort 1 were explored (Fig. 6a, b) (Supplementary Table 2). Five genes, *TSC22D3*, *PRF1*, *GIMAP7*, *KLF6* and *CXCR4*, were differentially expressed in all NK cell subsets of DHS patients compared with dapsone-tolerant individuals and healthy donors (Fig. 6a). Among these genes, *TSC22D3*, an immunoregulatory gene, was the most highly expressed DEG and showed a significant decrease in all NK cell subsets of DHS patients compared to dapsone-tolerant individuals (Fig. 6b). Unexpectedly, expression of *TSC22D3* in NK cells did not decrease after dapsone stimulation in both DHS patients and dapsone-tolerant individuals (*P* > 0.05, data not shown). These results suggest that the mRNA expression of *TSC22D3* is relatively stable and resistant to dapsone stimulation. The intrinsic difference of *TSC22D3* between DHS patients and dapsone-tolerant individuals could reflect a premorbid state that might determine the different function of NK cells and disease susceptibility.

To further explore the role of *TSC22D3*, *TSC22D3* knockout NK cell lines (NK-92MI) were established (Fig. 6c). Knockout of *TSC22D3* in NK-92MI resulted in decreased growth (Fig. 6d, e) and increased cell death (*P* < 0.05, unpaired t-test) (Fig. 6f). These findings are in alignment with the lower expression of *TSC22D3* in NK cells and frequency of NK cells in DHS patients compared to dapsone-tolerant individuals from cohort 1 (Figs. 1d and 6b). Additionally, knockout of *TSC22D3* enhanced NK-92MI activation, characterized by high secretion of effector molecules, including IFN-γ, perforin and GZMB (*P* < 0.05, unpaired t-test) (Fig. 6g). These results are consistent with the effector phenotype of *CD56*^high^ *XCL1/2*^high^ NK cell subsets in DHS patients but not in dapsone-tolerant individuals from cohort 2 (Fig. 4m, n). Collectively, these findings demonstrated that the effector function and proliferation of NK cells showed a mutual exclusion, which was mediated by the molecule of TSC22D3.

### NK cells with *TSC22D3* deficiency enhanced CD8+ T cell responses through both cytokine secretion and cell-cell-contact mechanisms

**Fig. 7:**
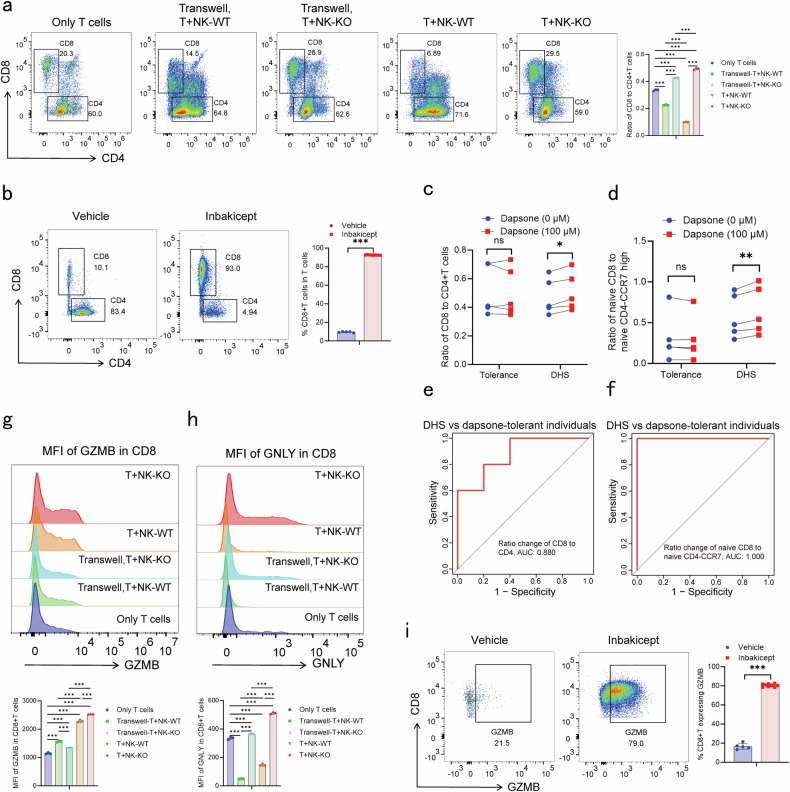
NK cells with *TSC22D3* deficiency enhanced CD8+ T cell response through both cytokines secretion and cell-cell-contact mechanisms. **a** Co-culture or trans-well assays were performed using 4 × 10^5 *TSC22D3* knockout or WT NK 92MI and 1 × 10^6 T cells from DHS patients in 24 well plate for three days. The ratio of CD8+ T/CD4+ T cells were calculated. One-way ANOVA followed by LSD was used for statistical analysis (***P < 0.001). **b** The frequency of CD8+ T cells in T cells from Fig. 5a were analyzed by flow cytometry. The unpaired two-sided student’s t-test was used for statistical analysis (***P < 0.001). **c**, **d** The ratio of total CD8+ T/CD4+ T cells (**c**) and naive CD8+ T/*CCR7*^high^ naive CD4+ T cells (**d**) from cohort 2 were calculated. e-f ROC curve showed the AUC, sensitivity and specificity of the ratio change of total CD8+ T/CD4+ T cells (**e**) and naive CD8+ T/*CCR7*^high^ naive CD4+ T cells (**f**) after dapsone stimulation in distinguishing DHS patients from dapsone-tolerant individuals. The paired two-sided student’s t-test was used for statistical analysis (*P < 0.05 and **P < 0.01). **g**, **h** The MFI of GZMB (**g**) and GNLY (**h**) in CD8+ T cells from a were analyzed by flow cytometry. One-way ANOVA followed by LSD was used for statistical analysis (***P < 0.001). **i** The percentage of CD8+ T cells that expressed GZMB from Fig. 5a were analyzed by flow cytometry. The unpaired two-sided student’s t-test was used for statistical analysis (***P < 0.001)

NK cells are involved in activation, proliferation and polarization of T cells.^14^ To investigate the role of NK cells with *TSC22D3* deficiency on T cells, trans-well assays comprising a co-culture of NK-92MI and T cells from DHS patients were performed. *TSC22D3* knockout NK cells increased the ratio of CD8+ T cells to CD4+ T cells through both cytokine secretion and cell-cell-contact mechanisms (*P* < 0.05, unpaired t-test) (Fig. 7a). In contrast, wild-type NK cells decreased the ratio of CD8+ T cells to CD4+ T cells through both cytokine secretion and cell-cell-contact (*P* < 0.05, unpaired t-test) (Fig. 7a). These results suggested *TSC22D3* deficiency leads to a shift of NK cells from promoting CD4+ T cells to CD8+ T cells proliferation. Furthermore, activating effector NK cell subsets in vivo by IL-15 superagonist also increased the percentage of CD8+ T cells in the mouse model (*P* < 0.05, t-test) (Fig. 7b). These results were consistent with the findings that both the ratio of total CD8+ T/CD4+ T cells and naive CD8+ T/*CCR7*^high^ naive CD4+ T cells increased in DHS patients but not in dapsone-tolerant individuals after dapsone stimulation (Fig. 7c–f).

In addition to the regulation of the percentage of CD8+ T cell, *TSC22D3* knockout and wild-type NK cells could directly activate CD8+ T cells as determined by increased expression of GZMB through both cytokine secretion and cell-cell-contact mechanisms (*P* < 0.05, unpaired t-test) (Fig. 7g and Supplementary Fig. 5a). Unexpectedly, *TSC22D3* knockout and wild-type NK cells showed opposite effects on regulation of the expression of GNLY in CD8+ T cells. *TSC22D3* knockout NK cells enhanced GNLY expression, while wild-type NK cells inhibited the expression in CD8+ T cells (*P* < 0.05, unpaired t-test) (Fig. 7h and Supplementary Fig. 5b). These results suggested that *TSC22D3* knockout NK cells have a more pronounced effect on CD8+ T cell effector function. Consistent with this in vitro experimental findings, activating effector NK cell subsets in vivo by IL-15 superagonist also activated CD8+ T cells as determined by increased expression of GZMB (Fig. 7i). Collectively, these results demonstrated *TSC22D3* determined the functional phenotype of NK cells, and activated effector NK cells have a positive regulation on CD8+ T cell activation.

## Discussion

Management of patients with SCARs represents a major challenge as disease mechanisms are not fully defined. The role of the adaptive immune system in SCARs is well established.^8,11,12,31–34^ However, the cooperative regulation of T cells in the development of SCARs by the innate immune system remains unexplored. In this study, sex-related aberrant blood NK cells are shown to be associated with the incidence of DHS. Among these NK cells, two kinds of functionally distinct subsets, *CD56*^high^ *XCL1/2*^high^ NK and *CD56*^low^ *XCL1/2*^low^, were found to regulate the reactivity and immunoregulation of DHS. These features were governed by the gene of *TSC22D3*, which showed a different expression between DHS patients and dapsone-tolerant individuals. More importantly, the NK cells with *TSC22D3* deficiency promoted CD8+ T cell responses and a shift from promoting proliferation of CD4+ T to CD8+ T cells, implying an important role for the innate immune system in regulating drug-induced allergic disease.

Evolutionarily conserved sex variables determine the outcome of diseases. In this study, we found the incidence of DHS in females was significant higher than in males. Our data are consistent with a recent case-control study that found females to be associated with a higher chance of dapsone-induced adverse drug events (odds ratio, OR = 3.61).^35^ Additionally, our meta-analysis showed females were more likely to develop other adverse drug reactions than males. Similar sex-related differences are observed in other allergic disease, such as allergic asthma and eczema^36^ and atopic dermatitis (AD).^37^ Sex-related differences in susceptibility to these diseases has been postulated to originate from the differential immune system composition. For example, it has been demonstrated that males express a higher percentage of NK cells than females.^19,20^ Consistent with these results, male DHS patients showed an increased expression of NK cells and a longer onset time for the development of DHS when compared to females. Moreover, the frequency of NK cells in PBMC showed a significantly positive correlation with onset time of DHS. These findings suggested that sex-related different composition of NK cells are involved in the pathogenesis of DHS.

Recent work has shown that alteration in NK cells frequencies and functions are associated with other allergic diseases.^13,15,38^ In the setting of AD, which is associated with aberrant allergic inflammation, patients displayed a global reduction in blood NK cells, however, their NK cells showed an enhanced activation state.^15^ This is consistent with another recent study, which found that increasing numbers of NK cells were accompanied by decreased effector function.^20^ In contrast, increased frequency of NK cells accompanied by expression of effector and immunomodulatory molecules were shown in PBMC of peanut allergic patients stimulated with peanut protein.^13^ Furthermore, a longitudinal cohort analysis revealed the accumulation of killer cell lectin like receptor K1 (*KLRK1*)^low^ NK cells in AD patients was linked to more severe AD and sensitivity to allergens.^38^ Similarly, DHS patients also showed a global reduction of NK cells in the circulation at the recovery stage but increase and activation in PBMC stimulated with dapsone in vitro. These findings help to highlight the complexity of NK cells in regulating the immune response of allergic diseases.

Studies over the past two decades have identified functionally distinct NK cell subsets^39^ based on the expression of cell-surface markers, such as of CD56, CD16, CD69, natural cytotoxicity receptors (NCRs).^13,15,39^ In this study, we identified two kinds of functionally distinct NK cell subsets, *CD56*^high^ *XCL1/2*^high^ and *CD56*^low^ *XCL1/2*^low^ NK cells. *CD56*^high^ *XCL1/2*^high^ NK cells of DHS patients highly expressed multiple effector molecules, such as *INFG*, *GZMB* and *GNLY* compared to dapsone-tolerant individuals at baseline state. Furthermore, *CD56*^high^ *XCL1/2*^high^ NK cell subsets of DHS patients exhibit a dapsone-dependent response by increasing their frequency and highly expressing effector molecules (GZMB) compared to dapsone-tolerant individuals after dapsone stimulation. These data indicate that *CD56*^high^ *XCL1/2*^high^ NK cell subsets are important effector cells in the pathogenesis of DHS. Due to the lack of TCR, the activation of *CD56*^high^ *XCL1/2*^high^ NK cell subsets is likely to be indirect, mediated by monocytes which display an up-regulated expression of IL-15 after dapsone stimulation in DHS patients but not in tolerant individuals (Supplementary Fig. 6). Whereas, other three *CD56*^low^ *XCL1/2*^low^ NK cell subsets showed an immunoregulatory phenotype. At recovery stage, DHS patients exhibited down-regulated expression of *CCL4L2* (involved in recruitment of T cells to enhance immunity)^29,30^ compared to dapsone-tolerant individuals, reflecting a negative feedback loop to maintain homeostasis. After dapsone stimulation, dapsone-tolerant individuals but not DHS patients displayed down-regulated expression of *CCL4L2*, highlighting a more immunoregulatory capacity to external stimulus for dapsone-tolerant individuals. Although a global reduction of NK cells was shown in DHS patients, it was *CD56*^low^ *XCL1/2*^low^ but not *CD56*^high^ *XCL1/2*^high^ NK cell subsets that displayed decreased expression. Collectively, the immune balance between these two kinds of NK cells influenced the development of DHS.

The differences in NK cell subset composition and/or function between DHS patients and dapsone-tolerant individuals prompted us to investigate the underlying molecular mechanism. An immunosuppressive mediator, *TSC22D3* was identified to be a critical molecular determinant of different NK cells. Interestingly, *TSC22D3* not only had an effect on promoting NK cells proliferation but also inhibited effector function of NK cells, which might explain the mutual exclusion of effector function and proliferation in immune cells.^20,40^ It is possible that enhanced effector function of NK cells resulted in activation-induced cell death.^15^ In support of this perspective, our data showed that enhanced effector function of NK cells after knockout of *TSC22D3* also display increased cell death. Furthermore, *TSC22D3* knockout NK cells promoted CD8+ T cell activation and lead to a shift from promoting proliferation of CD4+ T to CD8+ T cells. These results are consistent with previous studies that showed activated NK cells are involved in stimulation of the proliferation and polarization of T cells.^14^ A shift from CD4+ T to CD8+ T cell activation is known to be an important immune event for development of SCARs.^41,42^ For example, multiple studies have shown infiltration of predominantly CD8+ T lymphocytes in skin lesion of SCARs patients.^41,42^ Consistent with these findings, the ratio of CD8+ T to CD4+ T cells significantly increased in PBMC of DHS patients after dapsone stimulation in vitro. Taken together, these observations demonstrate that *TSC22D3* is a key molecular determinant for the differences of NK cells which influence the composition and function of T cells associating with the development of DHS.

While our study demonstrates that *TSC22D3*-associated NK cell differences modulate adaptive immunity in DHS, certain limitations should be noted. First, the transcriptomic changes observed during recovery may reflect either the premorbid state or a disease memory phenotype. However, the consistent *TSC22D3* expression in NK cells across both DHS patients and dapsone-tolerant individuals is unaffected by dapsone stimulation, suggesting it represents an inherent NK cell feature and reflecting the premorbid state. Second, although we identified *TSC22D3* as a key regulator of NK-mediated CD8+ T cell activation, its precise regulatory networks controlling CD8+ T cell function require further elucidation. Finally, future studies should incorporate NK cell subset depletion experiments using conditional knockout models or subset-specific strategies to confirm NK cell subsets’ role in SCARs pathogenesis.

Overall, this study demonstrates that *TSC22D3* determines the functional phenotype of NK cells, and activated effector NK cells have a positive regulation on CD8+ T cell activation, which creates a mechanistic link between the innate and adaptive immune system in patients with SCARs. We emphasized the importance of the innate immune system in the susceptibility of SCARs, which could be transformative in a broad set of other allergic disease or autoimmune diseases. Furthermore, in a departure from the current broad or targeted immunosuppressive therapeutic strategy targeting adaptive immune system for SCARs, our findings offer a new insight in which combined antagonists targeting both effector NK cells and T cells may provide therapeutic benefit.

## Materials and methods

### Ethics statement

All procedures involving the use of human samples were performed under protocols approved by the ethics committee of the Shandong Provincial Institute of Dermatology and Venereology, Shandong Academy of Medical Science (approval #20190314KYKT004). Informed written consent and permissions to collect blood was obtained from the participants. Animal protocols used in the study were approved by the Animal Ethics Committee of Shandong Provincial Institute of Dermatology and Venereology (W202403120415).

### Study design

We performed a two-stage scRNA-seq of PBMC from DHS patients and controls. The recovery cohort (cohort 1) included PBMC from 14 DHS patients carrying HLA-B*13:01 (Supplementary Table 1) in the recovery stage (after recovery for 21 to 180 months), nine dapsone-tolerant individuals carrying HLA-B*13:01 after taking dapsone for at least two year (Supplementary Table 3) and eight healthy donors without HLA-B*13:01. Both DHS patients and dapsone-tolerant individuals are leprosy patients with treatment of dapsone. The stimulation cohort (cohort 2) included PBMC from five DHS patients carrying HLA-B*13:01 and five dapsone-tolerant individuals carrying HLA-B*13:01 stimulated with or without dapsone (100 µM) for four days. A retrospective review of 61 DHS patients (Supplementary Table 4) was performed to analyze the correlation of the onset time of DHS with the age and the sex. For retrospective analysis of the incidence of DHS in male and female, we extracted the epidemiological data of leprosy patients between January 2008 and December 2012 from LEPMIS, national center for STD and leprosy control in China. The diagnosis of DHS was made based on the criteria proposed by Richardus and Smith.^43^ Meta-analysis was used to analyze the incidence of ADRs between male and female. The in vitro functional experiments were performed using PBMC from 14 DHS patients and nine dapsone-tolerant HLA-B*13:01+ individuals. The roles of effector NK cells in the development of SCARs was evaluated in established mice model of SCARs. The roles of *TSC22D3* in regulating the function of NK cells was performed in *TSC22D3* knockout NK-92MI cell lines.

### scRNA-seq and data analysis

The scRNA-seq and data analysis were conducted as previously described.^12^ Briefly, thawing PBMC or the stimulated PBMC with or without dapsone (100 µM) for four days were conducted for barcode labeling and library construction using the 10× chromium system (10× genomics). Constructed single-cell 5′ libraries were subsequently sequenced by the Illumina Novaseq 6000. The gene expression was normalized using the method “LogNormalize” in seurat. The t-distributed stochastic neighbor embedding (t-SNE) was employed for visualization. The ‘FindNeighbors’ and ‘FindClusters’ functions were used for clustering with a resolution of 0.1 for recovery cohort and 0.4 for stimulation cohort. The ‘FindAllMarkers’ function was used for identifying cluster-specific marker genes with a filter of log2 fold change (log2FC) > 0.585 and adjusted P values < 0.05. The annotation of clusters was based on the expression profiles of cluster-specific markers and canonical signature genes such as *CD3D*, *CD4*, *CD8A*, *CD14*, *CD19* and *CD56* (*NCAM1*). The analysis of DEGs between different groups was conducted using the ‘FindMarkers’ function with a filter condition of |log2FC | > 0.585 and adjusted *P*-values < 0.05.

### Knockout of *TSC22D3* in NK 92MI cell lines

Knockout (KO) NK 92MI cell lines of *TSC22D3* were constructed using CRISPR/Cas9 technology (gRNA-B1: AGAGGAGGTGGAGATCCTGAAGG) by cyagen biosciences. The DNA mutations were verified by sanger sequencing and the protein expression of GILZ (*TSC22D3* encode protein) was analyzed by flow cytometry.

### Generation of T cells from DHS patients

PBMC from DHS patients were stimulated with dapsone (100 µM) for 14 days to expand the dapsone responsive T cells. These T cells were activated using ImmunoCult™ Human CD3/CD28/CD2 T cell activator (STEMCELL Technologies, Canada) and expanded in medium supplemented with 10 ng/mL interleukin-2 (PEPROTECH, New Jersey, USA).

### Flow cytometry analysis

PBMC from DHS patients and dapsone-tolerant individuals were plated at a concentration of 1 × 10^6 cells per well in 24 well plate in 1 ml RPMI 1640 (ATCC) supplemented with 10% FBS (Gibco, USA), 1% penicillin and streptomycin (Gibco, USA) and 25 µg/mL transferrin (Sigma, Germany). These PBMC were stimulated with dapsone (100 µM) for six days. The expression of intracellular (GZMB and GNLY) and/or surface marker (CD3, CD4, CD8 and CD56) per sample were analyzed by flow cytometry.

Co-culture or trans-well assay were performed using 4 × 10^5 *TSC22D3* knockout or WT NK 92MI and 1 × 10^6 T cells from DHS patients in 24 well plate for three days. The expression of intracellular (GZMB and IFN-γ) and/or surface marker (CD3, CD4, CD8 and CD56) per sample were analyzed by flow cytometry.

LIVE/DEAD^TM^ Fixable Aqua Dead Cell Stain Kit (Invitrogen, USA) was used to exclude dead cells from analysis of flow cytometry.

### ELISPOT assay

3 × 10^3 NK 92MI or *TSC22D3* knockout NK 92MI cells were plated in 96 well ELISPOT (Dakewe Biotech Co., Ltd., Shenzhen, China) plate. These cells were cultured for one day, and the expression of IFN-γ, GZMB and perforin was detected by ELISPOT assay according to the manufacturer’s instructions (Mabtech, Nacka Strand, Sweden).

### Meta-analysis on the incidence of ADRs between male and female

Searches of PubMed databases were performed using the following search strategy:(‘sex’ AND ‘cutaneous adverse reactions’ AND ‘drug’), which yielded 178 articles between 1979 and 2024. Studies that contained the incidence of ADRs related to specific drugs between male and female were included in the meta-analysis. After literature screening, seven articles were eligible for inclusion in the meta-analysis. The odds ratio (OR) and random effects model were used in the meta-analysis. Statistical synthesis was carried out with the Meta-Analysis module of OnlineMeta (https://smuonco.shinyapps.io/Onlinemeta/).

### Mice model of SCARs

As previously published,^15,18^ C57BL/6 mice were injected intraperitoneally with 100 μl of 0.2 mg/ml inbakicept (IL-15 superagonist) (MCE, New Jersey, USA) on day one and day four to induce effector NK cells activation. C57BL/6 mice were injected subcutaneously with 100 μl of 1 mg/ml AZD5582 (antagonist of inhibitor of apoptosis proteins, IAP) (MCE, New Jersey, USA) on day one to induce SCARs like symptoms. 10% dimethyl sulfoxide in normal saline was used as vehicle control. Mice were euthanized on day five after AZD5582 injection and the sites were photographed and scored. And splenic cells and lesions samples were collected for in vitro analysis.

### Statistics

The “bimod” (Likelihood-ratio test) was used to analyze the DEGs in the scRNA-seq data and bonferroni was used to correct the P value. The chi-square test was used to analyze the incidence of DHS. Pearson correlation coefficient was used to performed correlation analysis. One-way ANOVA was used for all multi-group comparisons followed by least significant difference (LSD) for pairwise comparisons. Otherwise, unpaired or paired two-sided student’s t-test was used to analyze the other data, where indicated.

## Supplementary information

Supplementary_Materials

Table S2

## Data Availability

All data generated or used during the study appear in the submitted article. The raw sequence data reported in this paper have been deposited in the Genome Sequence Archive in National Genomics Data Center, China National Center for Bioinformation / Beijing Institute of Genomics, Chinese Academy of Sciences that are publicly accessible at https://ngdc.cncb.ac.cn/gsa-human (HRA010805, HRA000847 and HRA005913).
