# Supplementary material for: TSC22 domain family member 3 links natural killer cells to CD8+ T cell-mediated drug hypersensitivity
Source: Signal Transduct Target Ther. 2025 Jun 21;10:196. doi: 10.1038/s41392-025-02300-0 (PMC12182574; doi:10.1038/s41392-025-02300-0)
Supplement: Supplementary file 1 — Supplementary_Materials [file 41392_2025_2300_MOESM1_ESM.docx]

Supplementary Materials for

TSC22 domain family member 3 links natural killer cells to CD8+ T cell-mediated drug hypersensitivity

Lele Sun; Pengcheng Huai; Zhenzhen Wang; Qing Zhao; Yingjie Lin; Tingting Liu; Xiaotong Xue; Suiting Ao; Jiabao You; Yonghu Sun; Zihao Mi; Joshua Gardner; Paul J Thomson; Dean J Naisbitt; Xiaoli Meng; Jianjun Liu; Hong Liu; Furen Zhang

Correspondence to: Furen Zhang [(zhangfuren@hotmail.com)](mailto:(zhangfuren@hotmail.com)) or Hong Liu (hongyue2519@hotmail.com)

**This PDF file includes:**

Figures. S1 to S6

Tables S1, S3 and S4


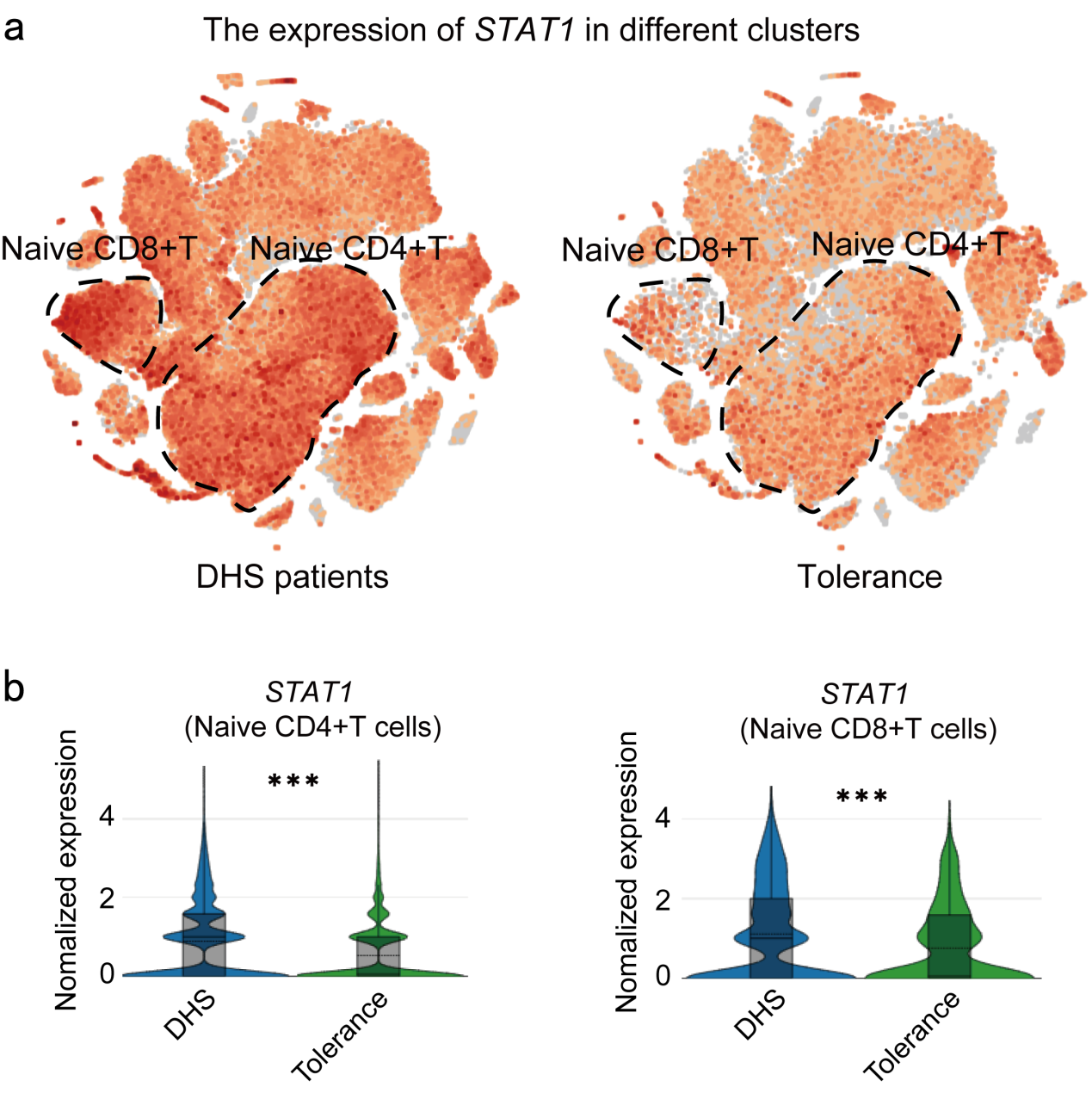


Figure. S1.

**The expression of *STAT1* in naive CD4+T and naive CD8+T cells.** **a** t-SNE and **b** violin plots show the expression of *STAT1* in naive CD4+T and naive CD8+T cells. The "bimod" (Likelihood-ratio test) and bonferroni corrected were used to analyze the data of the scRNA-seq data (***P < 0.001).

**
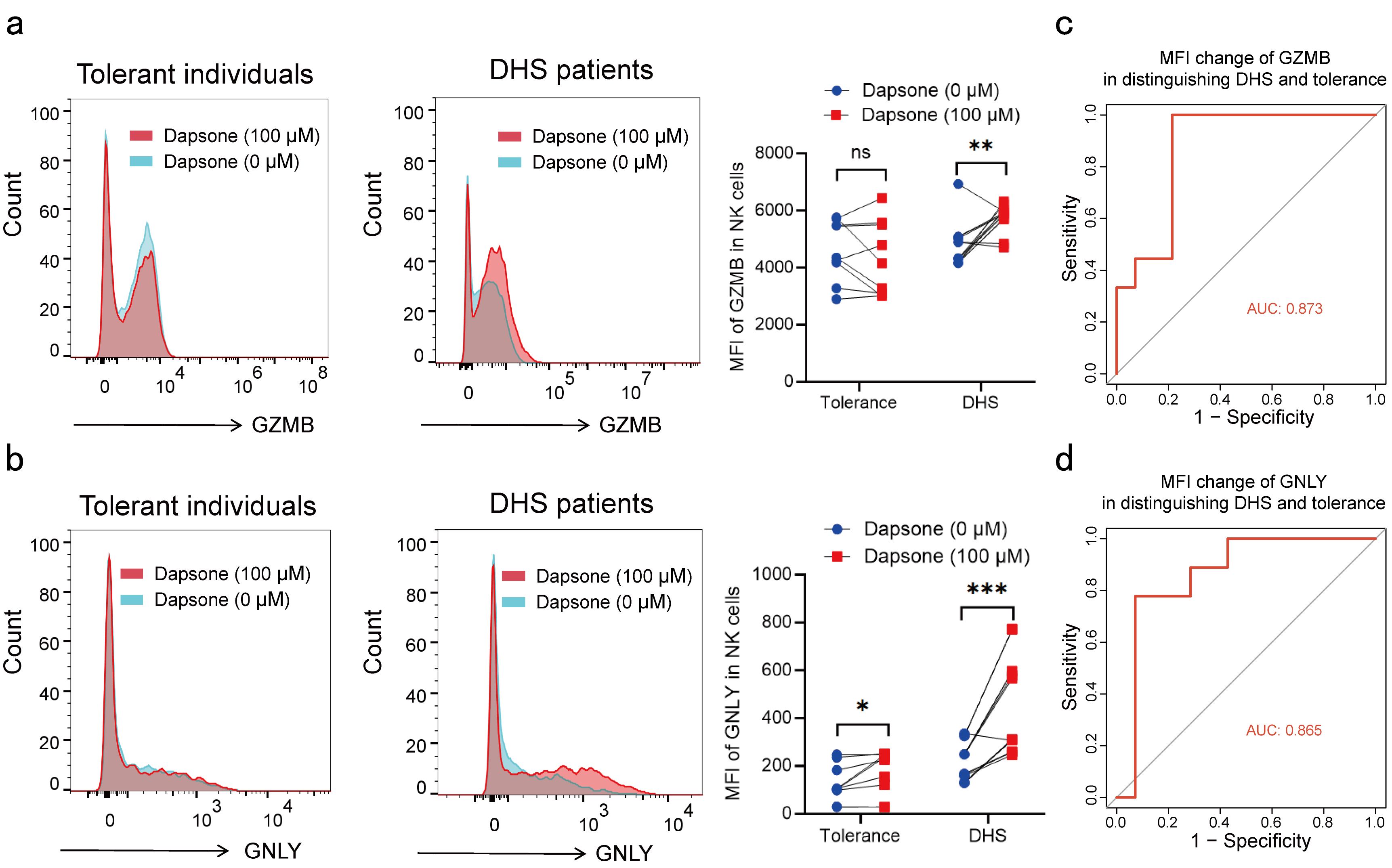
Figure. S2.**

**The dapsone dependent response in NK cells was greater in DHS patients than daspone tolerant individuals.** PBMC from 14 DHS patients and nine dapsone tolerant individuals were cultured with or without dapsone (100 µM) for six days, and the MFI of GZMB (**a**) and GNLY (**b**) in NK cells were analyzed by flow cytometry analysis. The paired two-sided student’s t-test was used for statistical analysis (*P < 0.05,**P < 0.01 and ***P < 0.001). ROC curve showed the AUC, sensitivity and specificity of MFI change of GZMB (**c**) and GNLY (**d**) in NK cells after dapsone stimulation in distinguishing DHS patients from dapsone-tolerant individuals.


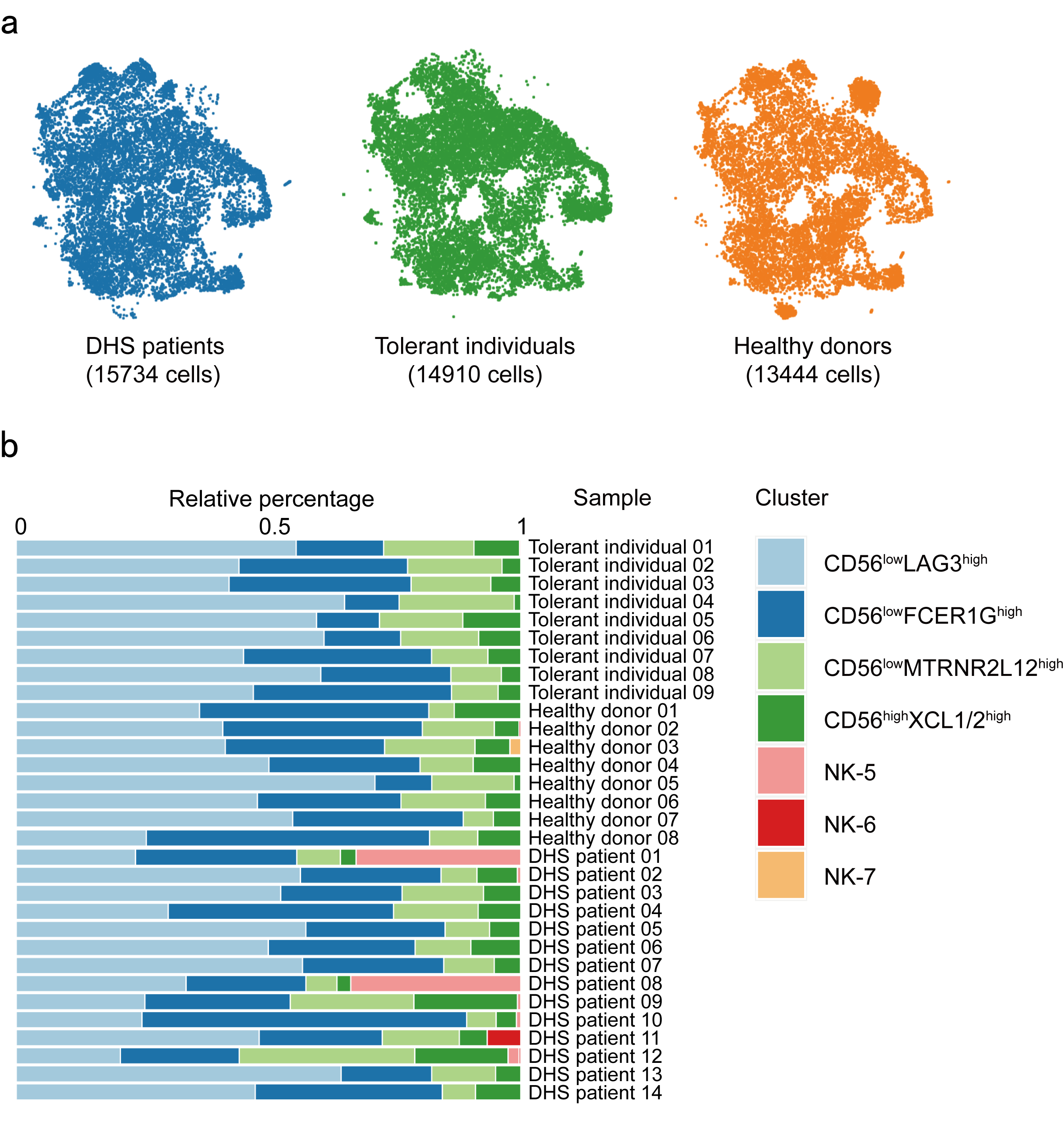
**Figure. S3.**

**Sub-clustering analysis for NK cells from cohort 1. a** t-SNE visualization displaying 15734 cells from DHS patients, 14910 dapsone-tolerant individuals and 13444 healthy donors, respectively. **b** Relative percentage of each NK cell subset in total PBMC in each sample.


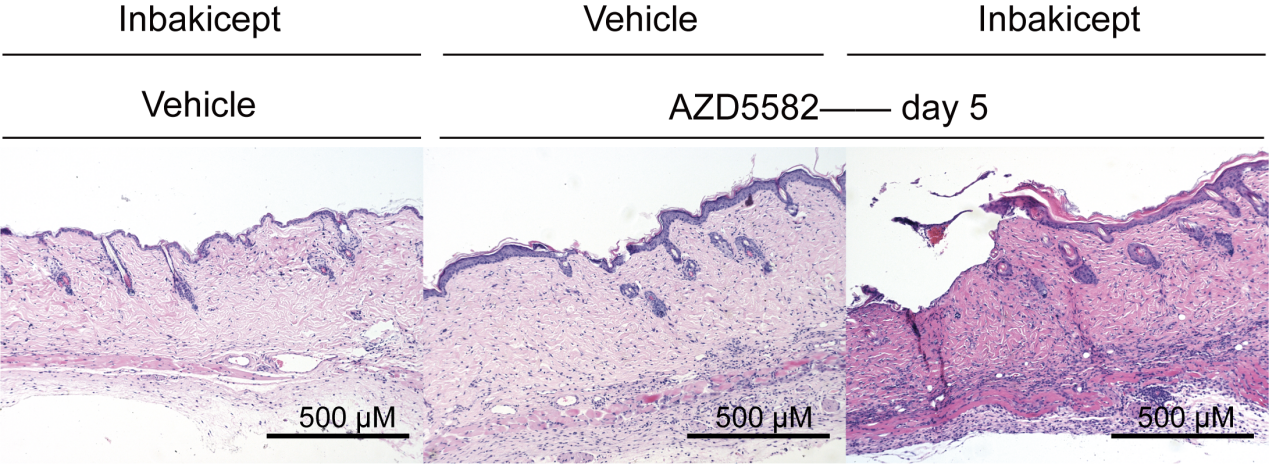
**Figure. S4.**

**Effector NK cells aggravated the SCARs like symptoms in established mice model of SCARs.** C57BL/6 mice were injected subcutaneously with 100 μl of 1 mg/ml AZD5582 on day one and injected intraperitoneally with 100 μl of 0.2 mg/ml inbakicept (IL-15 superagonist) on day one and day four. On day five, mice were euthanized and lesions samples were collected for histology analysis.


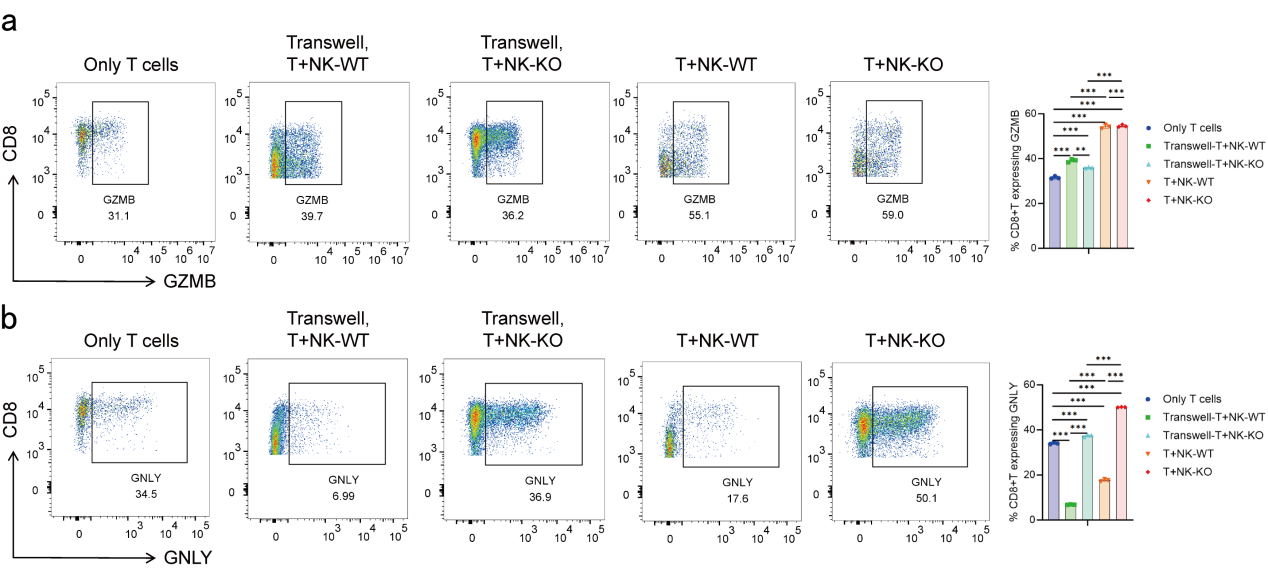
**Figure. S5.**

**NK cells with *TSC22D3* deficiency enhanced CD8+ T cell response through both cytokines secretion and cell-cell-contact mechanisms.** Co-culture or transwell assay were performed using 4×10^5 *TSC22D3* knockout or WT NK 92MI and 1×10^6 T cells from DHS patients in 24 well plate for three days. The percentage of CD8+ T cells that expressed GZMB **(a)** and GNLY **(b)** were analyzed by flow cytometry. One-way ANOVA followed by LSD was used for statistical analysis (**P < 0.01 and ***P < 0.001).


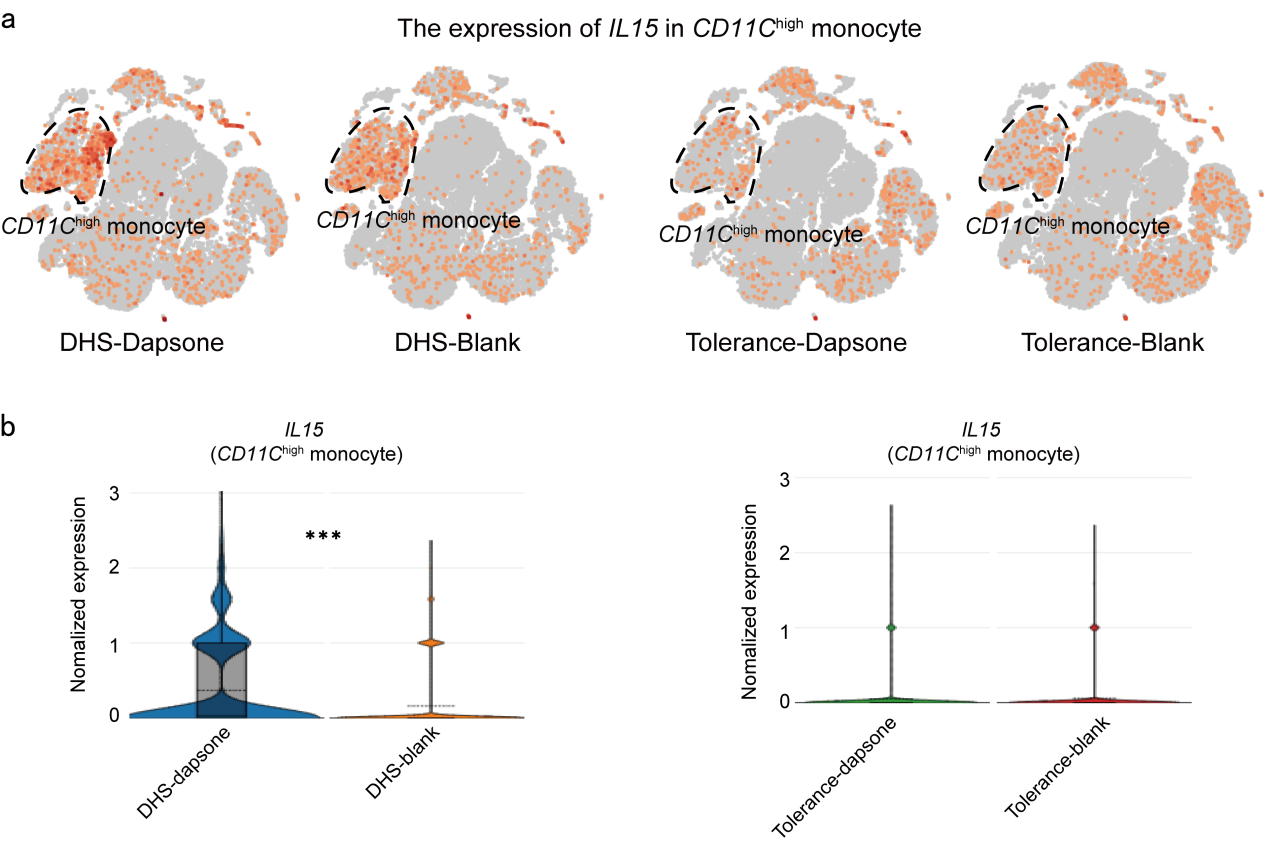
**Figure. S6.**

**The expression of *IL15* in *CD11C*^high^ monocyte. a** t-SNE and **b** violin plots show the expression of *IL15* in *CD11C*^high^ monocyte of DHS patients and tolerant individuals stimulated with dapsone or medium (blank). The "bimod" (Likelihood-ratio test) and bonferroni corrected were used to analyze the data of the scRNA-seq data (***P < 0.001)

Table S1.

| **Table S1: The clinical characteristics, ELISPOT and patch testing of the DHS patients in cohort 1 and cohort 2.** | | | | | | | | | | | | |
| --- | --- | --- | --- | --- | --- | --- | --- | --- | --- | --- | --- | --- |
| No. | Age | Gender | Onset time^☯^ (days) | Clinical Manifestation# | | | | | ELISPOT | | | Patch Test |
|  |  |  |  | Fever | Generalized Rash | Lymphadenopathy | Hepatic function abnormalities | jaundice | IFN-γ | IL-5 | Granzyme B |  |
| 1* | 34 | M | 23 | √ | √ | √ | √ | √ | + | + | + | - |
| 2 | 22 | F | 21 | √ | √ | √ | √ |  | - | + | + | + |
| 3* | 31 | F | 17 | √ | √ | √ | √ |  | + | + | + | + |
| 4 | 22 | M | 37 | √ | √ |  | √ |  | + | + | - | - |
| 5 | 42 | M | 35 | √ | √ | √ | √ |  | + | + | + | / |
| 6 | 44 | F | 20 | √ | √ |  | √ |  | - | - | - | + |
| 7* | 40 | M | 30 | √ | √ |  | √ |  | + | + | + | + |
| 8* | 41 | M | 48 | √ | √ | √ | √ |  | + | + | + | + |
| 9 | 54 | M | 16 | √ | √ |  | √ |  | - | + | + | - |
| 10 | 42 | M | 22 | √ | √ |  | √ |  | - | - | + | - |
| 11 | 30 | F | 26 | √ |  | √ | √ |  | - | - | + | - |
| 12 | 55 | M | 18 | √ | √ | √ | √ |  | + | + | + | - |
| 13* | 41 | F | 14 | √ | √ | √ | √ |  | - | - | - | + |
| 14 | 28 | M | 51 | √ | √ |  | √ |  | + | + | + | + |
| Note: ☯, the onset time after administration of dapsone; #, The DHS was diagnosed based on the criteria proposed by Richardus and Smith; *, The DHS patients were used in cohort 2; √, The patients presented this symptom. | | | | | | | | | | | | |

Table S3.

| **Table S3: The clinical characteristics of nine dapsone tolerant individuals.** | | | | | |
| --- | --- | --- | --- | --- | --- |
| No. | Age | Gender | Disease | Duration of taking dapsone  (Months) | Interval time☯  (Months) |
| 1 | 34 | M | Leprosy | 24 | 24 |
| 2 | 42 | F | Leprosy | 24 | 84 |
| 3 | 54 | F | Leprosy | 24 | 106 |
| 4 | 77 | M | Leprosy | 24 | 522 |
| 5 | 65 | F | Leprosy | 30 | 42 |
| 6 | 69 | F | Leprosy | 24 | 450 |
| 7 | 67 | F | Leprosy | 40 | 535 |
| 8 | 83 | M | Leprosy | 37 | 629 |
| 9 | 23 | M | Leprosy | 24 | 24 |
| Note: ☯, Interval between drug withdrawal and blood sample collection | | | | | |

Table S4.

| **Table S4: The clinical characteristics of 61 DHS patients.** | | | | | | | |
| --- | --- | --- | --- | --- | --- | --- | --- |
| No. | Age | Gender | Onset time^☯^ (days) | No. | Age | Gender | Onset time^☯^ (days) |
| 1 | 49 | F | 35 | 32 | 26 | M | 22 |
| 2 | 38 | F | 45 | 33 | 26 | M | 33 |
| 3 | 33 | F | 32 | 34 | 20 | M | 41 |
| 4 | 32 | F | 13 | 35 | 19 | M | 27 |
| 5 | 30 | F | 34 | 36 | 19 | M | 29 |
| 6 | 26 | F | 22 | 37 | 16 | M | 42 |
| 7 | 21 | F | 18 | 38 | 14 | M | 31 |
| 8 | 17 | F | 29 | 39 | 14 | M | 20 |
| 9 | 15 | F | 30 | 40 | 13 | M | 61 |
| 10 | 37 | F | 21 | 41 | 26 | M | 44 |
| 11 | 26 | F | 32 | 42 | 38 | M | 9 |
| 12 | 38 | F | 23 | 43 | 43 | M | 35 |
| 13 | 43 | F | 33 | 44 | 29 | M | 36 |
| 14 | 31 | F | 15 | 45 | 38 | M | 31 |
| 15 | 54 | F | 35 | 46 | 17 | M | 33 |
| 16 | 42 | F | 37 | 47 | 21 | M | 30 |
| 17 | 11 | F | 24 | 48 | 42 | M | 26 |
| 18 | 59 | F | 1 | 49 | 31 | M | 30 |
| 19 | 40 | F | 31 | 50 | 54 | M | 32 |
| 20 | 25 | F | 4 | 51 | 54 | M | 18 |
| 21 | 55 | M | 34 | 52 | 71 | M | 35 |
| 22 | 54 | M | 36 | 53 | 41 | M | 32 |
| 23 | 49 | M | 43 | 54 | 34 | M | 49 |
| 24 | 46 | M | 34 | 55 | 30 | M | 31 |
| 25 | 39 | M | 22 | 56 | 70 | M | 39 |
| 26 | 36 | M | 41 | 57 | 25 | M | 36 |
| 27 | 35 | M | 18 | 58 | 21 | M | 26 |
| 28 | 33 | M | 47 | 59 | 50 | M | 18 |
| 29 | 31 | M | 31 | 60 | 42 | M | 36 |
| 30 | 30 | M | 43 | 61 | 42 | M | 31 |
| 31 | 30 | M | 40 |  |  |  |  |
| Note: ☯, the onset time after administration of dapsone. | | | | | | | |
